# Management of Hydrofluoric Acid Burns

**Published:** 2014-10-27

**Authors:** Netanel Alper, Kunj Desai, Sidney Rabinowitz

**Affiliations:** ^a^Division of Plastic Surgery, Department of Surgery, Rutgers New Jersey Medical School, Newark, NJ; ^b^Hackensack University Medical Center, Hackensack, NJ

**Keywords:** burns, calcium gluconate, chemical burns, fingertip necrosis, hydrofluoric acid

**Figure:**
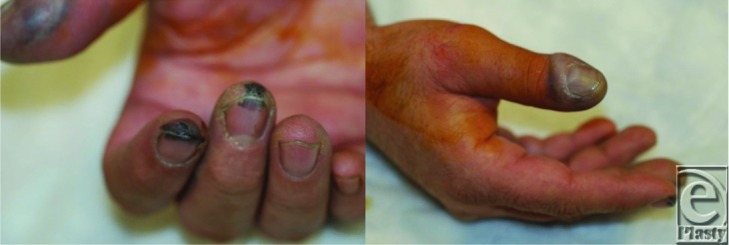


## DESCRIPTION

This is a 45-year-old male who injured himself while working on a motor vehicle 5 days prior to presentation. A cleaning agent containing hydrofluoric acid came into contact with both hands. Initially the patient only complained of minor discomfort but over the course of 5 days, he developed progressively worsening pain and swelling involving the right thumb and left index finger. He also noted other similar areas on both hands.

## QUESTIONS

1. **What are the common sources of hydrofluoric acid that contribute to injuries?**
2. **How does hydrofluoric acid cause injury?**
3. **How do hydrofluoric acid burns present?**
4. **How should hydrofluoric acid burns be managed?**

## DISCUSSION

Hydrofluoric acid (HFA) is an inorganic acid that has widespread use in both industry and home. It is utilized in the glass industry to etch and frost glass; the semiconductor industry to dissolve silica; and more extensively in the automobile industry for rust removal and aluminum cleaning. Industrial HFA solutions range from 20% to 70% concentration. Hydrofluoric acid is also available in lower concentrations (<12%) as a rust-removal agent, automobile wheel cleaner, and as a heavy-duty domestic cleanser.^1^^,^^2^

Hydrofluoric acid consists of protein hydrolyzing hydrogen ions and fluoride ions. The hydrogen ions cause corrosion, coagulation necrosis, and eschar formation. The severity of which is dependent upon the concentration of the solution and the damage is typically superficial. However, the fluoride ions are responsible for the majority of injury associated with HFA burns. They are highly lipophilic and can penetrate deep into tissue, resulting in liquefactive necrosis that is associated with excruciating pain. They are strong scavengers of bivalent cations like calcium and magnesium. The degree of calcium binding can be so severe that it may cause systemic hypocalcemia resulting in muscle cramping, weakness, and cardiac effects. In addition, the disruption of cell membrane Na+/K+ ATPase pumps can cause a concomitant hyperkalemia in addition to hypocalcemia and hypomagnesemia.^1^^,^^3^^,^^4^

The concentration of HFA and the duration of exposure determine the extent of injury. Concentrations greater than 50% cause immediate injury, while concentrations between 20% and 50% can take several hours before becoming apparent. Products that have concentrations less than 20% can take as much as 24 hours for injury to develop. If untreated, deep tissue destruction can continue for several days after the initial exposure. Hands and digits are the most frequently injured part of the body (HFA can penetrate latex gloves therefore vinyl or rubber gloves are safer options). Initially, injured dermal tissue becomes edematous and blanches and is accompanied by severe pain that is often out of proportion to the physical appearance of the burn. Eschar formation may occur as well, depending on the concentration. As the damage to deeper tissue worsens over time, gray skin patches that represent underlying areas of tissue necrosis may be seen. Subungual tissue may also be affected. It is important to monitor the patient's cardiac status particularly QT interval prolongation on EKG (Electrocardiogram) that can portend potentially fatal arrhythmias.^1^^,^^3^

Treatment begins with removing any contaminated clothing along with copious irrigation of the exposed skin for 15 to 30 minutes. Any blisters that may have formed should be unroofed to remove any fluoride ions contained in the fluid within. After irrigation, application of a topical 2.5% to 5% calcium gluconate (CG) gel to the affected area should help to neutralize the free fluoride ions by providing an alternative source of calcium. This can be prepared by adding 3.5 to 7 g of CG powder to a 5-ounce tube of any water-soluble lubricant.^5^ Putting a surgical glove on the patient's hand after application of the gel helps to ensure sustained therapeutic effects. The best marker of successful treatment is relief of pain. If pain is alleviated, gel applications should continue every 4 to 6 hours for about 3 to 4 days. If pain persists despite gel treatment, any of the following may be attempted: intralesional injection of CG (for small burns), regional infusion of a CG solution by means of the Bier block (for larger lesions); or intra-arterial infusions. The last option is reserved for severe burns that do not respond to any other treatment methods. Local anesthetic agents via a digital or regional block are highly effective at managing pain but do not treat the actual injury.^1^^,^^6^ The patient above presented with tissue necrosis on multiple digits of both hands. After extensive debridement and treatment follow-up images show excellent recovery.

## Figures and Tables

**Figure 1:**
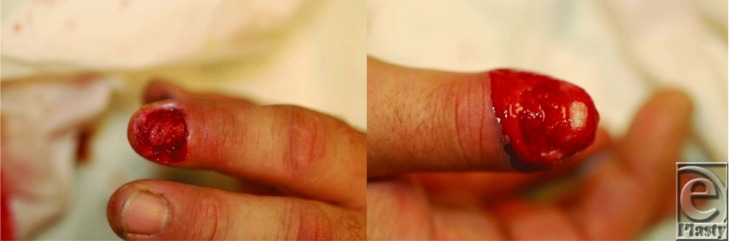
After debridement and nail removal.

**Figure 2:**
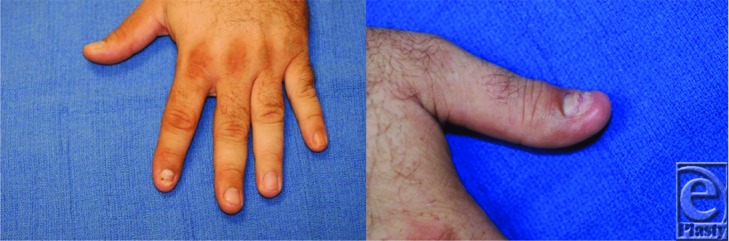
Follow-up visit, showing good healing.
